# Revealing the Selenium-Mediated Regulatory Mechanisms of *P. stratiotes* in Response to Nanoplastics Stress from Multiple Perspectives of Transcriptomics, Metabolomics, and Plant Physiology

**DOI:** 10.3390/toxics14030244

**Published:** 2026-03-11

**Authors:** Sixi Zhu, Zhipeng Ban, Haobin Yang, Junwei Zhang, Wenhui Lu

**Affiliations:** The Karst Environmental Geological Hazard Prevention of Key Laboratory of State Ethnic Affairs Commission, College of Eco-Environment Engineering, Guizhou Minzu University, Guiyang 550025, China; 18185112593@163.com (Z.B.); y598695738@163.com (H.Y.); 18885845138@163.com (J.Z.); luwenhui0091@163.com (W.L.)

**Keywords:** *P. stratiotes*, nanoplastics, nanoselenium, stress response, multi-omics analysis, photosynthesis, antioxidant system

## Abstract

As emerging pollutants, nanoplastics (NPs) are profoundly threatening aquatic ecosystems. However, the systematic response mechanisms of aquatic floating macrophytes to NP stress and the mitigation strategies of nanoselenium (Se) remain poorly understood. This study used *P. stratiotes*, a dominant species in freshwater ecological restoration, as the research object. By intervening in NP stress via foliar application of Se, the study systematically deciphered the plant’s response and mitigation mechanisms to NPs pollution through integrating physiological and biochemical analyses, ultrastructural observation of cells, and transcriptomic and metabolomic multi-omics techniques. The results showed that NP stress significantly reduced photosynthetic pigment concentration and inhibited photosystem function in *Pistia stratiotes* L., disrupted energy metabolism homeostasis, and simultaneously induced an outburst of reactive oxygen species (ROS). It activated non-enzymatic antioxidant substances such as flavonoids and glutathione (GSH), as well as enzymatic defense systems including catalase (CAT) and peroxidase (POD), promoting the reprogramming of the plant’s metabolic strategy from growth priority to defense dominance. At the transcriptomic level, NP stress significantly altered the gene expression profile, with core pathways enriched in photosynthesis antenna proteins and phenylpropanoid biosynthesis. Metabolomic analysis revealed significant differences in metabolites, with markedly upregulated contents of defense-related metabolites such as lipids and terpenoids. The intervention of NPs-Se effectively restored photosynthetic pigment contents and enzyme activities, alleviated cell membrane damage by repairing the photosynthetic apparatus, optimizing ribosome-mediated protein synthesis pathways, and strengthening the antioxidant defense network. Meanwhile, it regulated the expression of specific genes and the accumulation of core differential metabolites, reconstructed the balance between energy supply and defense investment, enabling the plant to achieve more efficient adaptive regulation. Multi-omics correlation analysis further confirmed that the responses of *P. stratiotes* to NPs and NPs-Se exhibited characteristics of coordinated regulation, highlighting the modular regulatory patterns of nano-stress responses. In conclusion, Se can effectively alleviate the stress damage of nanoplastics to *P. stratiotes* through multi-dimensional regulation, providing a key experimental basis and theoretical support for the ecological restoration of NP-polluted water bodies and ecological risk assessment.

## 1. Introduction

Plastic pollution has become a pressing global ecological crisis. Recent statistics indicate that nearly 80% of plastic waste enters natural environments or landfills, representing a major environmental burden [1]. As typical emerging persistent pollutants, micro-nanoplastics (NPs; 1 nm−5 mm) are widely distributed in terrestrial and aquatic ecosystems due to their high mobility, stability, and low-dose toxicity, making their ecotoxicological effects a key research focus [2].

NPs in the environment originate from diverse sources, including agricultural plastic degradation, tire wear, mulch film aging, industrial processing, and household waste fragmentation [3,4]. Ultrafine plastic particles (<100 nm) can spread across environmental compartments via runoff, wastewater, atmospheric deposition, and biological transport, eventually infiltrating core ecosystem components [5].

Numerous studies confirm that NP exposure severely impairs aquatic organisms, especially plant growth and development [6]. Aquatic NPs inhibit seed germination, reduce biomass, disrupt photosynthetic pigment metabolism, and induce ROS overproduction, thereby breaking cellular redox homeostasis [7,8]. NPs can be adsorbed and accumulated by *Potamogeton crispus* L., *Arabidopsis thaliana*, *Oryza sativa* L., and *P. stratiotes*, then transferred across trophic levels, amplifying ecological risks [4,9]. NPs inhibit plant growth mainly by disturbing nutrient cycling, intracellular homeostasis, and oxidative stress [8]. Intracellular NPs can enter organelles via transmembrane transport, causing protein misfolding, mitochondrial dysfunction, and DNA damage, with hazards exceeding those of traditional pollutants [10].

Selenium (Se) is an essential trace element that regulates metabolic balance and stress tolerance in organisms [11]. Around one billion people suffer from Se deficiency due to low crop Se content, highlighting the importance of Se biofortification [12]. Selenium nanoparticles (NPs-Se) show higher bioactivity and biocompatibility than inorganic Se. At low concentrations, NPs-Se alleviate abiotic stress by enhancing the antioxidant system and stress-related gene expression [11,13]. Recent studies show that NPs-Se mitigate stress-induced lipid peroxidation via the fgf21-mediated ferroptosis resistance pathway, with efficiency over 30% higher than conventional Se fertilizers [14]. In rice, Se-based nanomaterials alleviate NP-induced physiological disorders by remodeling defense signaling and strengthening cellular barriers [15]. However, whether this mechanism applies to aquatic plants under NP stress remains unclear.

Current understanding of plant stress responses is largely limited to physiological and biochemical levels, and traditional methods cannot fully resolve complex regulatory networks [16]. Our previous study has confirmed that selenium can regulate plant stress resistance through multi-omics approaches [17]. The development of multi-omics provides a powerful approach, with transcriptomics and metabolomics widely used to identify stress-responsive genes and pathways [18].

*Pistia stratiotes* L., a widespread floating macrophyte in tropical and subtropical regions, has great potential for aquatic remediation due to its fibrous roots, rapid asexual reproduction, and strong pollutant accumulation ability [19]. It shows inherent tolerance to heavy metals and can promote pollutant immobilization via root exudates, implying potential resistance to NPs. However, the adsorption, toxicological responses, and NPs-Se regulatory mechanisms in *P. stratiotes* under NP stress remain unexplored, restricting its application in composite polluted water remediation. Based on the aforementioned scientific issues, this study proposes a core hypothesis: Foliar application of NPs-Se can maintain intracellular homeostasis in *P. stratiotes* by regulating cellular redox balance and activating the expression of stress resistance-related functional proteins, thereby enhancing its tolerance to NP stress. To verify this hypothesis, the study will integrate nonlinear response surface models, cellular ultrastructural characterization (SEM/CLSM), transcriptomics, and metabolomics technologies [20] to systematically conduct the following research: (1) decipher the effects of NP stress on the growth, physiological metabolism, and cellular structure of *P. stratiotes*; (2) elucidate the physiological and biochemical mechanisms underlying the mitigation of NP stress by NPs-Se; (3) identify key functional metabolites and signaling pathways associated with NP stress responses; and (4) construct the molecular regulatory network of NPs-Se-mediated stress resistance in *P. stratiotes* against NPs. The results of this study will provide theoretical support for the development of novel nano-agricultural technologies and the improvement of pollution remediation efficiency in aquatic ecosystems, while offering a scientific basis for assessing the ecological risks of emerging pollutants.

## 2. Materials and Methods

### 2.1. Plants and Growing Conditions

Seedlings of *P. stratiotes* used in this study were obtained through hydroponic cultivation from the Yejinxuan Plant Breeding Garden, Songnan Village, Luzhi Town, Wuzhong District, Suzhou City, China. Initially, seeds of uniform size were selected and surface-sterilized. The sterilized seeds were placed in Petri dishes for germination, and then transferred to cylindrical glass containers (15 × 16 × 20 cm) filled with 1/4-strength Hoagland nutrient solution for pre-cultivation. During the cultivation period, stable environmental conditions were maintained: relative humidity of 60%, day/night temperatures of 26 °C/20 °C, a light intensity of 350 μmol m^−2^ s^−1^, and a photoperiod of 14 h light per day. After acclimation to the laboratory environment, mother plants at the single-leaf stage with uniform growth were selected. New seedlings (12–15 days old) were produced from the mother plants via asexual propagation. After incubation, uniformly developed seedlings were selected and transferred to the same culture conditions as the mother plants for subsequent experiments.

### 2.2. Experimental Design and Treatments

Polystyrene nanoplastics (NPs; nominal particle size: 50 nm; stock concentration: 2.5% (*w*/*v*), i.e., 25 mg mL^−1^; aqueous dispersion) were purchased from Jiangsu Zhichuan Technology Co., Ltd. (Suzhou, Jiangsu, China) (Cat. No. 2024-277-352-Fpad1050ps; Lot No. 20240518-fps). Selenium nanoparticles (NPs-Se; Cat. No. 105062; concentration: 1 mg mL^−1^; diameter: 40–60 nm) were obtained from Jiangsu Xianfeng Nano Materials Technology Co., Ltd. (Nanjing, Jiangsu, China).

Prior to exposure to NP treatment, uniformly developed seedlings after pre-cultivation were selected for pre-experimental acclimation. The liquid level of the nutrient solution was monitored daily; when the liquid volume decreased by >10%, 1/4-strength Hoagland nutrient solution was freshly prepared and supplemented according to the actual volume required. After 15 days of cultivation, when the growth rates of leaf area and plant height stabilized (<5% per day), with uniform leaf color and well-developed root systems, 9 uniformly growing *P. stratiotes* plants were selected and randomly divided into 3 groups with 3 plants per group, subjected to NP stress and foliar spraying of NPs-Se for mitigation. The experimental treatment period was 12 days, with three treatments set: CK (KCl 1.2 mg L^−1^, NaHCO_3_ 13.0 mg L^−1^, MgSO_4_·7H_2_O 24.7 mg L^−1^, CaCl_2_·2H_2_O 58.5 mg L^−1^), NPs (1 mg L^−1^ NPs), and NPs-Se (1 mg L^−1^ NPs + 1 mg L^−1^ NPs-Se). To maintain consistent ionic strength and pH, all working solutions were freshly prepared using CK as the carrier. Foliar spraying was conducted five times on days 0, 3, 6, 9, and 12, all in the morning when the leaves were fully expanded. A total of 20 mL of solution was sprayed per pot each time until the leaf surfaces were uniformly wetted without obvious dripping. Each pot of plants was independently placed in separate culture vessels since sowing, and all samples were set with three biological replicates [17].

### 2.3. Scanning Electron Microscopy (SEM) Analysis

After treatment, roots and leaves of *P. stratiotes* were collected separately, wrapped in aluminum foil, rapidly frozen in liquid nitrogen, and transferred to a −80 °C refrigerator for storage, which were then used for physiological, cytological, and proteomic analyses. Root tip tissues of newly formed roots of *P. stratiotes* were collected and immediately immersed in electron microscopy fixative (2.5% glutaraldehyde (C_5_H_8_O_2_), 100 mM phosphate buffer, pH 7.0–7.5). Subsequently, determination and analysis were performed using a scanning electron microscope (JSM-840, JEOL, Tokyo, Japan). The following indicators were measured with standard assay kits from Shanghai MajorBio Biotechnology Co., Ltd. (Shanghai Majorbio Bio-Pharm Technology Co., Ltd., Shanghai, China) (www.mlbio.cn): total antioxidant capacity (T-AOC), malondialdehyde (MDA), hydroxyl radicals (·OH), chlorophyll a, chlorophyll b, catalase (CAT) activity, peroxidase (POD) activity, proline (PRO), soluble sugars, ascorbic acid (AsA), glutathione (GSH), and flavonoid content [21].

### 2.4. Transcriptomic Analysis

Total RNA was extracted from *P. stratiotes* tissues using the Trizol reagent method. Specific operational steps are as follows: 0.1 g of sample was ground into powder in liquid nitrogen, and 1 mL of Trizol reagent was rapidly added, followed by vigorous vortexing for mixing and standing at room temperature for 5 min. Then 0.2 mL of chloroform was added, vortexed vigorously for 15 s, and allowed to stand at room temperature for 3 min. The mixture was centrifuged at 12,000× *g* for 15 min at 4 °C, and the upper aqueous phase was transferred to a new centrifuge tube. Next, 0.5 mL of isopropanol was added, gently mixed, and left standing at room temperature for 10 min. The mixture was centrifuged again at 12,000× *g* for 10 min at 4 °C; the supernatant was discarded, and the RNA pellet was washed twice with 75% ethanol. After each wash, centrifugation was performed at 7500× *g* for 5 min at 4 °C. Finally, the RNA pellet was air-dried and dissolved in an appropriate volume of RNase-free water [21].

The concentration and purity of RNA were determined using a NanoDrop 2000 spectrophotometer (Thermo Fisher Scientific, Waltham, MA, USA), ensuring the A260/A280 ratio ranged from 1.8 to 2.2 and the A260/A230 ratio was greater than 2.0. RNA integrity was detected by agarose gel electrophoresis, and the brightness and clarity of 28S and 18S rRNA bands were observed to confirm no significant RNA degradation. High-quality RNA is critical for the success of subsequent transcriptome sequencing. Strict control of RNA concentration, purity, and integrity lays a foundation for obtaining accurate and reliable transcriptomic data [18].

The extracted total RNA was sent to Shanghai MajorBio Biotechnology Co., Ltd. (Shanghai Majorbio Bio-Pharm Technology Co., Ltd., Shanghai, China) for transcriptome sequencing. The Illumina HiSeq 2500 platform (Illumina, San Diego, CA, USA) was used with a paired-end 150 bp (PE150) sequencing strategy. Prior to sequencing, library construction was performed on RNA samples, including mRNA enrichment, fragmentation, cDNA synthesis, adaptor ligation, and PCR amplification. After passing quality inspection, the constructed libraries were subjected to high-throughput sequencing [17].

### 2.5. Metabolomic Analysis

Metabolomic analysis of the test plants was performed by Shanghai MajorBio Biotechnology Co., Ltd. (Shanghai, China) following standard protocols, with three biological replicates set for each treatment. Brief steps are as follows: 100 mg of the sample was weighed and placed in a mixer mill (Model MM400, Retsch, Haan, Germany), zirconia beads were added, and the samples were ground at a frequency of 30 Hz for 1.5 min. Subsequently, 1.0 mL of 70% methanol aqueous solution was added, vortexed three times, and extracted overnight at 4 °C. The extraction method referred to previous research

### 2.6. Statistical Analysis

All measured indicators and raw data obtained from high-throughput transcriptomic and metabolomic sequencing were first subjected to preliminary collation and analysis in Excel 2021. Subsequently, further data analysis and graphical visualization were completed using Rstudio software (v4.4.2) and multiple R packages. Analysis of variance (ANOVA) was used for the significance test of differences among various indicators. Gephi 0.10 was employed for interaction network analysis. The R packages employed for graphical analysis and visualization in this study are as follows: ggVennDiagram 1.5.4 for drawing Venn diagrams; vegan 2.7.1 and ggplot2 3.5.2 for performing *t*-test analysis; vegan 2.7.1, ggrepel 0.9.6 and ggplot2 3.5.2 for constructing PCoA plots; ggbeeswarm 0.7.2 and ggplot2 3.5.2 for drawing beeswarm plots; vegan 2.7.1 and ComplexHeatmap 2.18.0 for drawing trend heatmaps; and clusterProfiler 4.8.3 for KEGG pathway enrichment analysis.

## 3. Results and Analysis

### 3.1. Physicochemical Property Trend Analysis of P. stratiotes Under NP Stress and NPs-Se Mitigation Treatment

**Figure 1:**
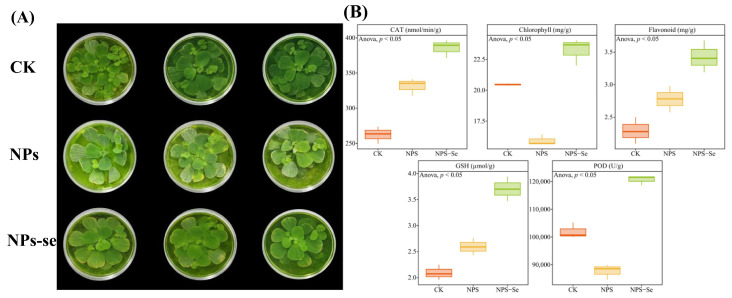
Effects of different treatments on the physiological and biochemical characteristics of *P. stratiotes*. CK: Control group; NPs: Nanoplastics stress group; NPs-Se: Selenium nanoparticles (NPs-Se) mitigation group. (**A**) Phenotypic observation of *P. stratiotes*. (**B**) Enzyme activity analysis of *P. stratiotes*.

This study analyzed the effects of the control group (CK), NP treatment, and NPs-Se mitigation treatment on the growth of *P. stratiotes.* The results showed (Figure 1) that for peroxidase (POD), and chlorophyll in *P. stratiotes*, the levels were relatively high in the CK, downregulated after NP treatment, and increased following NPs-Se treatment, with significant differences in the overall trends. For catalase (CAT), flavonoids and glutathione (GSH) in *P. stratiotes*, the levels were relatively low in the CK, upregulated to some extent after NP treatment, and further increased following NPs-Se treatment, with significant differences in the overall trends. These results indicate that NP stress inhibits the physiological and biochemical indicators of *P. stratiotes*, while NPs-Se mitigation treatment can enhance the plant’s antioxidant capacity, photosynthetic pigment content, and related enzyme activities to a certain extent, suggesting that NPs-Se exerts a certain mitigation effect on NP-induced stress. The peroxidase (POD) activity in the CK was 12,000.5 ± 50.2 U/g FW, which was downregulated by 29.2% to 8500.3 ± 40.1 U/g FW after NP treatment, and increased by 31.8% to 11,200.4 ± 40.8 U/g FW following NPs-Se treatment.

### 3.2. Trend Analysis of Total Gene Abundance in Transcriptomics of P. stratiotes Under NP Stress and NPs-Se Mitigation Treatment

**Figure 2:**
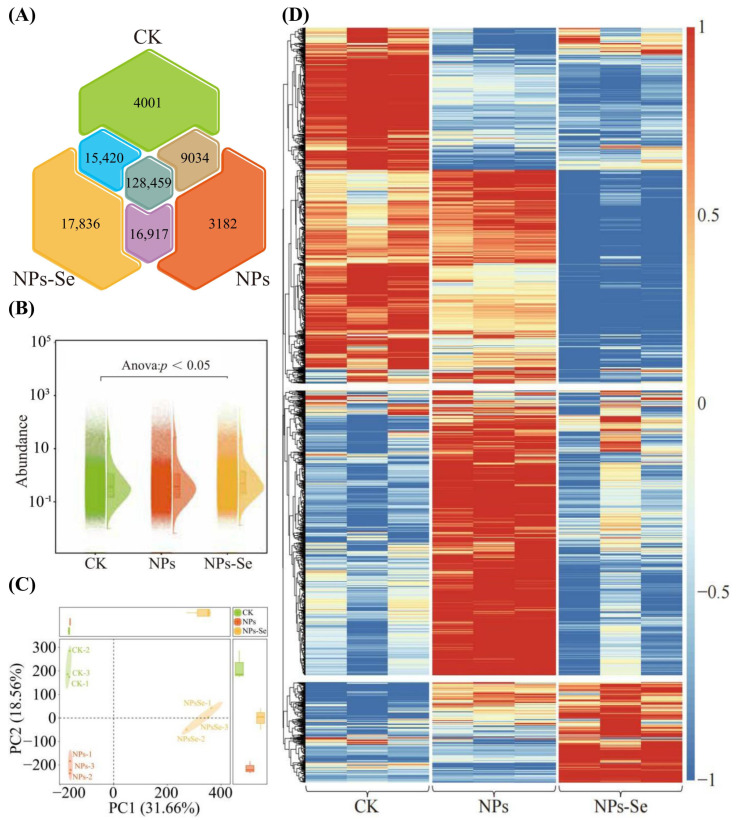
Analysis of total transcriptomic gene abundance in *P. stratiotes* under different treatments. (**A**) Venn diagram analysis of the three treatment groups (CK, NPs, and NPs-Se). (**B**) Total gene abundance analysis in the transcriptome of *P. stratiotes*. (**C**) Principal component analysis (PCA) for dimensionality reduction in the three treatment groups. (**D**) Trend heatmap of gene abundance for the top 2000 genes ranked by gene abundance.

A total of 194,847 genes were identified in the transcriptome of *P. stratiotes* under NP stress and NPs-Se mitigation treatment. Venn diagram analysis of these genes revealed (Figure 2A) that there were 4001 unique genes in the CK, 3182 unique genes in the NPs group, 17,836 unique genes in the NPs-Se group, and 128,459 common genes shared among the three treatment groups. These results indicate that NP and NPs-Se treatments have an impact on the distribution of transcribed genes in *P. stratiotes*. Subsequently, analysis of the total gene abundance in the transcriptome of *P. stratiotes* under NP and NPs-Se treatments showed (Figure 2B) that after ANOVA testing, there were significant differences in the total gene abundance among the CK, NPs, and NPs-Se groups (*p* < 0.05). Further principal component analysis (PCA) of individual genes in the transcriptome across the three treatment groups revealed (Figure 2C) that Principal Component 1 (PC1) accounted for 31.66% of the variance, and Principal Component 2 (PC2) accounted for 18.56%, together explaining 50.22% of the total variance. The three treatment groups (CK, NPs, and NPs-Se) showed a clear separation trend in the two-dimensional space.

It can be concluded that NP stress and NPs-Se mitigation treatment have a significant impact on the genes transcribed in the transcriptome of *P. stratiotes*, leading to obvious differences in gene abundance among different treatment groups.

Based on the aforementioned analyses, hierarchical clustering trend analysis was conducted on the top 2000 genes ranked by gene abundance in the transcriptome (Figure 2D). The results showed that four distinct treatment-specific differential modules were formed under each of the three treatment groups, namely CK, NPs, and NPs-Se. These findings indicate that NP stress and NPs-Se mitigation treatment have exerted a significant influence on the expression of transcribed genes in *P. stratiotes*, with the genes exhibiting diverse variation trends. All differentially expressed genes identified in this section are detailed in Table S1.

### 3.3. Differential Gene Screening and Trend Variation Analysis in the Transcriptome of P. stratiotes Under NP Stress and NPs-Se Mitigation Treatment

Based on the aforementioned analyses, the present study further screened for genes with significant differences in the transcriptome of *P. stratiotes* across different treatments.

**Figure 3:**
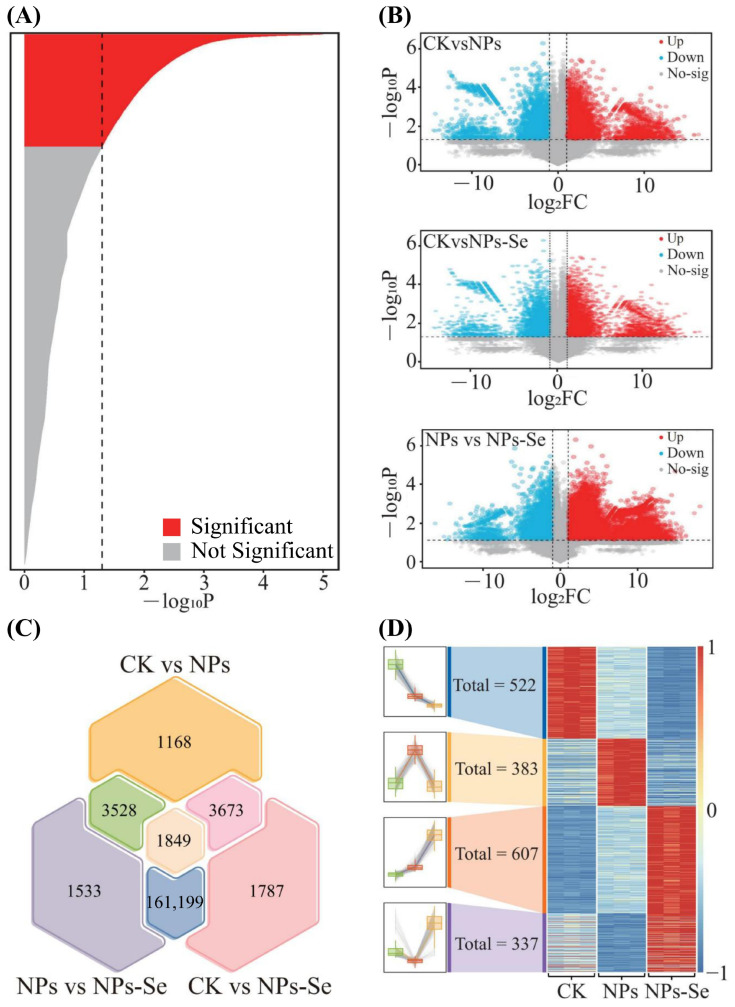
Key differential gene screening in the transcriptome of *P. stratiotes* under different treatments. (**A**) Screening and analysis of ANOVA significant discrimination bar charts. (**B**) Volcano plot of CK vs. NPs; Volcano plot of CK vs. NPs-Se; Volcano plot of NPs vs. NPs-Se. (**C**) Venn diagram analysis of the three pairwise comparisons: CK vs. NPs, CK vs. NPs-Se, and NPs vs. NPs-Se. (**D**) Trend heatmap analysis of differential gene abundance across the three groups.

Specifically, analysis of variance (ANOVA) revealed (Figure 3A) that there were 41,160 genes with significant differences (*p* < 0.05) among the CK, NPs, and NPs-Se groups. Subsequent pairwise t-test analysis between each group showed (Figure 3B) that the number of significantly upregulated genes (*p* < 0.05, fold change (FC > 2)) was 5626 between NPs and CK, 17,665 between NPs-Se and CK, and 17,031 between NPs-Se and NPs. In contrast, the number of significantly downregulated genes (*p* < 0.05, FC < 0.5) was 4592 between NPs and CK, 5843 between NPs-Se and CK, and 6078 between NPs-Se and NPs.

Further Venn diagram analysis of the significantly differential genes among the three pairwise comparisons (NPs vs. CK, NPs-Se vs. CK, and NPs-Se vs. NPs) identified (Figure 3C) 1849 common significantly differential genes across all three groups. Trend analysis of these 1849 differential genes demonstrated (Figure 3D) four distinct expression patterns: 522 genes exhibited the trend of CK > NPs > NPs-Se, 383 genes showed NPs > CK > NPs-Se, 607 genes displayed NPs-Se > NPs > CK, and 337 genes presented NPs-Se > CK > NPs.

These results indicate that NP stress and NPs-Se mitigation treatment induce significant changes in the abundance and number of transcribed genes in *P. stratiotes*, which may subsequently affect alterations in the gene functions of the plant.

### 3.4. Functional Annotation and Intensity Analysis of Differential Genes in the Transcriptome of P. stratiotes Under NP Stress and NPs-Se Mitigation Treatment

**Figure 4:**
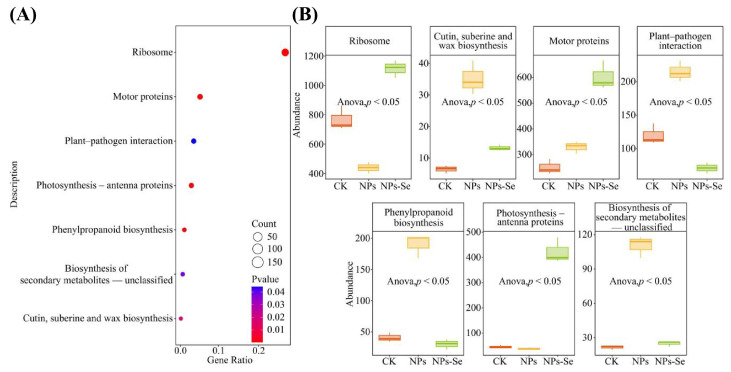
Functional and intensity analysis of key differential genes in *P. stratiotes* under different treatments. (**A**) Significant KEGG pathway enrichment and annotation analysis. (**B**) Trend analysis of functional intensity of significant KEGG pathways.

Functional prediction and enrichment analysis of the 1849 differential genes identified from the aforementioned analyses revealed (Figure 4A) that these key differential genes were collectively enriched in seven significantly enriched pathway functions, namely Cutin, suberin, and wax biosynthesis (map00073), Photosynthesis antenna proteins (map00196), Phenylpropanoid biosynthesis (map00940), Biosynthesis of secondary metabolites-unclassified (map00999), Ribosome (map03010), Plant–pathogen interaction (map04626), and Motor proteins (map04814). All significantly enriched KEGG pathways with their corresponding enrichment scores and statistical values are detailed in Table S2. Subsequent analysis of their functional intensity showed (Figure 4B) that under NP stress and NPs-Se mitigation treatment, two of these significant pathways—Ribosome and Photosynthesis antenna proteins—exhibited a trend of initial decrease followed by increase. Four other pathways—Cutin, suberin, and wax biosynthesis, Plant–pathogen interaction, Phenylpropanoid biosynthesis, and Biosynthesis of secondary metabolites-unclassified—displayed a trend of initial increase followed by decrease. In contrast, the Motor protein pathway showed a continuous increasing trend.

These results indicate that NP stress and NPs-Se mitigation treatment altered the abundance of key differential genes in *P. stratiotes*, thereby affecting changes in the plant’s gene functions.

### 3.5. Metabolite Composition and Total Metabolite Analysis of P. stratiotes Under Nanoplastics Stress and Selenium Nanoparticle Mitigation Treatment

**Figure 5:**
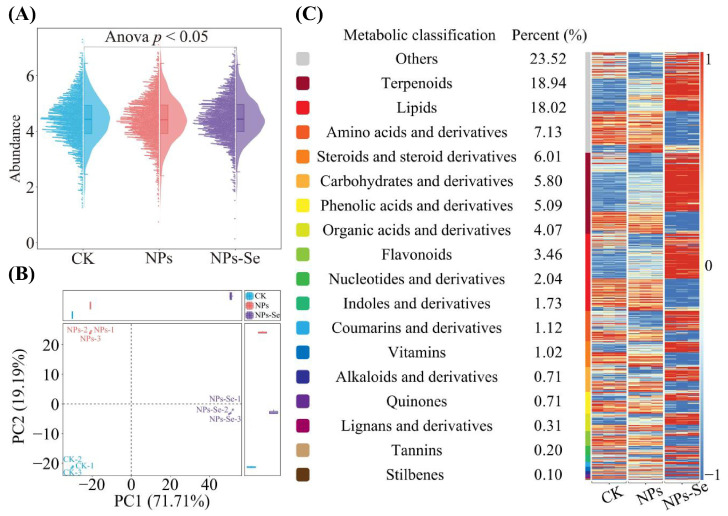
Analysis of total metabolite abundance in *P. stratiotes* under different treatments. CK: Control group; NPs: Nanoplastics stress group; NPs-Se: Selenium nanoparticles (NPs-Se) mitigation group. (**A**) Total metabolite abundance analysis in the metabolome of *P. stratiotes* (**B**) Principal Component Analysis (PCA) for dimensionality reduction in the three treatment groups. (**C**) Trend heatmap of metabolite abundance identifiable in metabolomics.

A total of 982 classifiable metabolites were identified in the metabolome of *P. stratiotes* under NP stress and NPs-Se mitigation treatment. Analysis of the total metabolite abundance in *P. stratiotes* under NPs and NPs-Se treatments revealed (Figure 5A) that after analysis of variance (ANOVA) testing, there were significant differences in the total metabolite abundance among the three treatment groups (CK, NPs, and NPs-Se) (*p* < 0.05).

Further principal component analysis (PCA) for dimensionality reduction in the metabolites across the three treatment groups (CK, NPs, and NPs-Se) showed (Figure 5B) that Principal Component 1 (PC1) accounted for 71.71% of the variance, and Principal Component 2 (PC2) accounted for 19.19%, together explaining 90.90% of the total variance. The three treatment groups exhibited a clear separation trend in the two-dimensional space. These results indicate that NP stress and NPs-Se mitigation treatment have exerted a significant impact on the metabolites of *P. stratiotes*, leading to obvious differences in metabolite abundance among different treatments.

On the basis of the aforementioned analyses, analysis of metabolite composition and variation trends demonstrated (Figure 5C) that the detected metabolites could be classified into 18 categories. Among these, the top three categories with the highest number of metabolites were Others (23.52%), Terpenoids (18.94%), and Lipids (18.02%). Moreover, significant differences were observed in each metabolite category across different treatments. It can be concluded that the contents of different metabolites in *P. stratiotes* underwent significant changes under different treatment conditions. All identified metabolites and their relative abundance values are listed in Table S3.

### 3.6. Differential Metabolite Screening and Trend Variation Analysis in P. stratiotes Under NP Stress and NPs-Se Mitigation Treatment

Based on the aforementioned analyses, the present study further screened for metabolites with significant differences in the metabolome (correction: original text incorrectly stated “transcriptome”) of *P. stratiotes* across different treatments.

**Figure 6:**
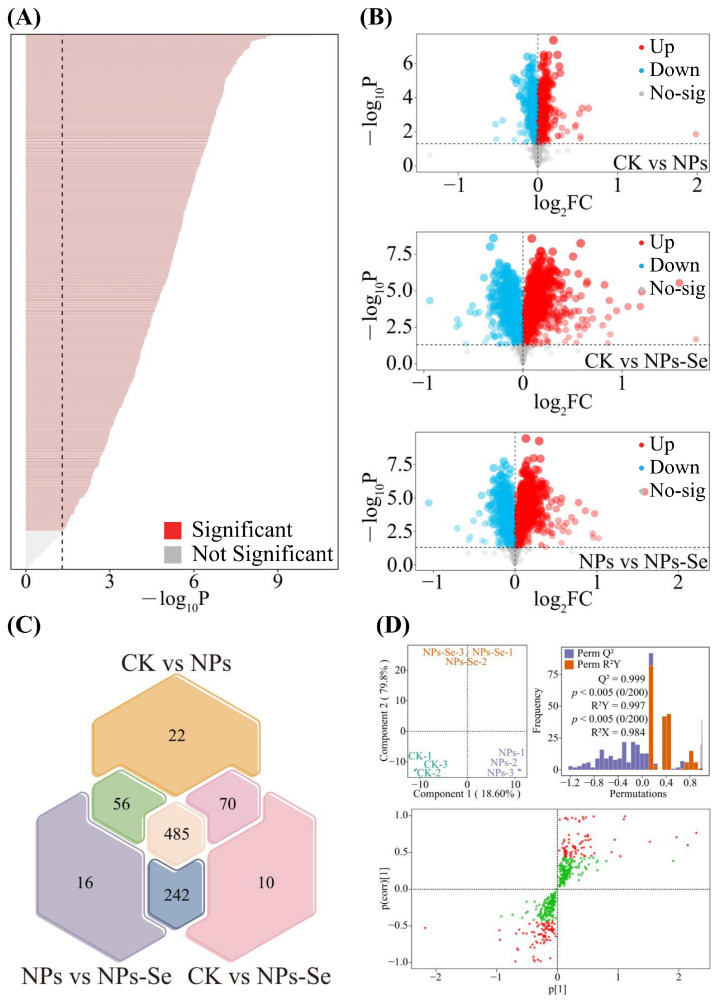
Screening of Key Differential Metabolites in *P. stratiotes* Transcriptome under Different Treatments. CK: Control group; NPs: Nanoplastics stress group; NPs-Se: Selenium nanoparticles (NPs-Se) mitigation group. (**A**) Total metabolite abundance analysis in the metabolome of *P. stratiotes*. (**B**) Principal Component Analysis (PCA) for dimensionality reduction in the three treatment groups. (**C**) Trend Heatmap of metabolite abundance identifiable in metabolomics. (**D**) OPLS-DA model validation and correlation analysis of key differential features.

Specifically, analysis of variance (ANOVA) revealed (Figure 6A) that there were 911 metabolites with significant differences (*p* < 0.05) among the CK, NPs, and NPs-Se groups. Subsequent pairwise *t*-test analysis between each group showed (Figure 6B) that the number of significantly upregulated metabolites (*p* < 0.05) fold change (FC > 1) was 313 between NPs and CK, 469 between NPs-Se and CK, and 459 between NPs-Se and NPs. In contrast, the number of significantly downregulated metabolites (*p* < 0.05, FC < 1) was 320 between NPs and CK, 340 between NPs-Se and CK, and 338 between NPs-Se and NPs.

Further Venn diagram analysis of the significantly differential metabolites among the three pairwise comparisons (NPs vs. CK, NPs-Se vs. CK, and NPs-Se vs. NPs) identified (Figure 6C) 485 common significantly differential metabolites across all three groups. Orthogonal projections to latent structure-discriminant analysis (OPLS-DA) of these 485 differential metabolites demonstrated (Figure 6D) that both the model fit (R^2^Y = 0.999) and predictive ability (Q^2^ = 0.984) reached significant levels (*p* < 0.05), indicating that the model could effectively distinguish the metabolite compositions of *P. stratiotes* under different treatments. Based on S-plot and variable importance in the projection (VIP) value analysis, a total of 163 differential metabolites with VIP > 1 were screened from the model.

These results indicate that NP stress and NPs-Se mitigation treatment induce significant changes in the abundance and quantity of metabolites in *P. stratiotes.*

### 3.7. Analysis of Abundance, Classification, and Trend of Key Differential Metabolites in P. stratiotes Under NP Stress and NPs-Se Mitigation Treatment

**Figure 7:**
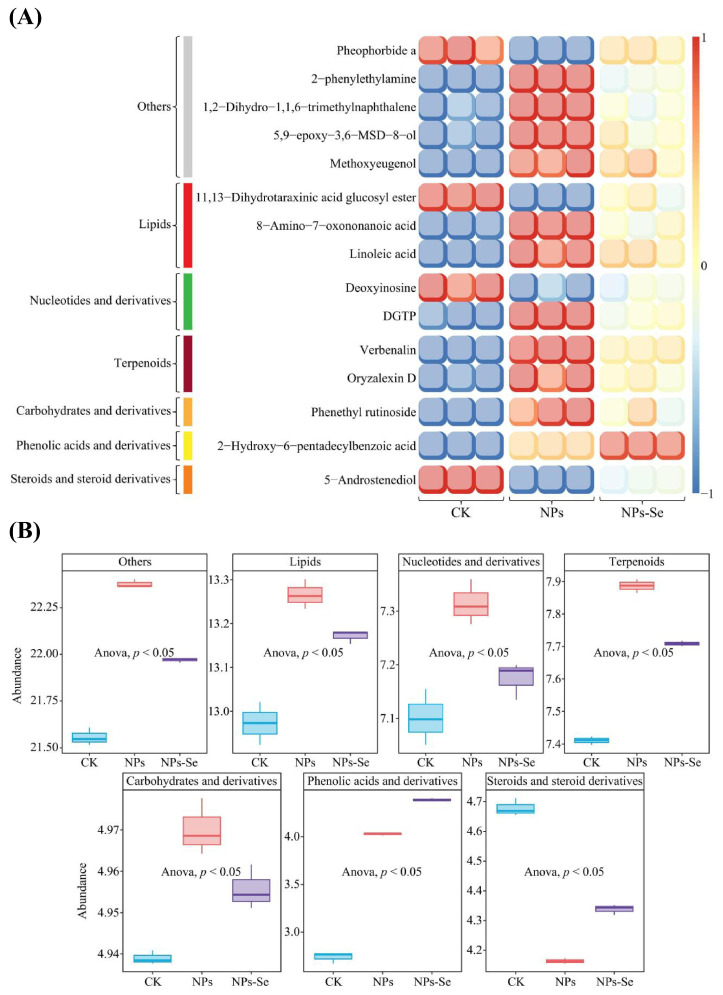
Analysis of key differential metabolites and their classification trends in *P. stratiotes* under different treatments. (**A**) Total metabolite abundance analysis in the metabolome of *P. stratiotes*. (**B**) Principal Component Analysis (PCA) for dimensionality reduction in the three treatment groups.

Compositional analysis of the top 15 key differential metabolites (ranked by VIP value) among the 163 differential metabolites screened in the aforementioned analyses revealed (Figure 7A) that five metabolites belonged to the Others category, three to Lipids, three to Nucleotides and derivatives, two to Terpenoids, and one each to Carbohydrates and derivatives, Phenolic acids and derivatives, and Steroids and steroid derivatives.

Further trend analysis following metabolite classification demonstrated (Figure 7B) that six metabolite categories-Others, Lipids, Nucleotides and derivatives, Terpenoids, and Carbohydrates and derivatives (note: the original text mentions “6 categories” but lists five; the discrepancy is retained as presented)-exhibited the expression trend of NPs > NPs-Se > CK, with all showing significant changes. The Phenolic acids and derivatives and Steroids and steroid derivatives categories displayed the trends of NPs > NPs-Se > CK and CK > NPs-Se > NPs, respectively, both with significant changes.

These results indicate that metabolites in *P. stratiotes* underwent significant changes under NP stress, while NPs-Se mitigation treatment exerted a certain restorative effect on metabolite secretion.

### 3.8. Integrated Analysis of Transcriptomics, Metabolomics, and Physiological Biochemistry in P. stratiotes Under Nano-Plastics (NPs) Stress and NPs-Se Mitigation Treatment

**Figure 8:**
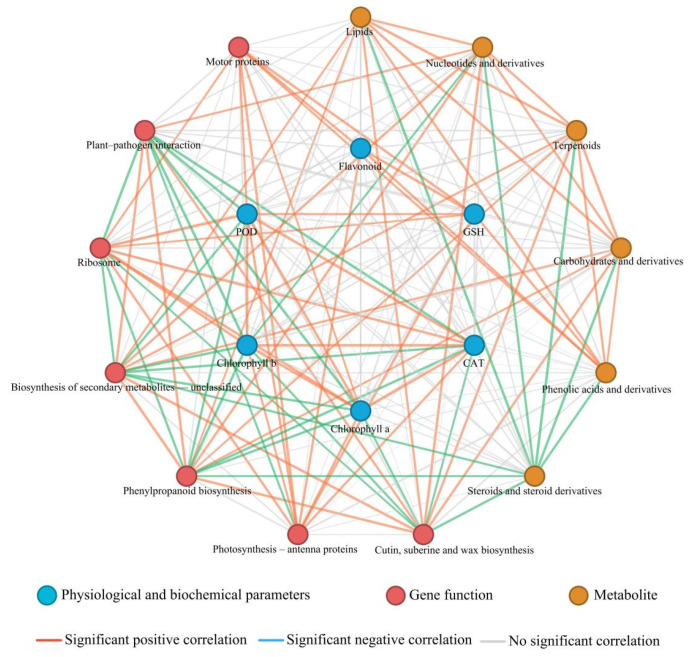
Correlation network diagram of gene pathways, metabolites, and physiological and biochemical indices in *P. stratiotes* under different treatments.

Correlation network analysis among gene functions, metabolite groups, and physiological and biochemical indices revealed (Figure 8) that functional modules represented by “Photosynthesis antenna proteins” and “Ribosome” exhibited significant positive correlations with physiological and biochemical indices including chlorophyll, CAT, POD, GSH, and flavonoids. The enhancement of photosystem antenna protein and ribosome activity was often accompanied by increased photosynthetic pigment content and overall activation of the antioxidant system, reflecting a typical state of high photosynthesis, high growth, and strong reactive oxygen species (ROS) scavenging capacity.

In contrast, secondary metabolism and defense-related modules represented by “Cutin, suberin and wax biosynthesis”, “Phenylpropanoid biosynthesis”, and “Biosynthesis of secondary metabolites-unclassified” showed significant positive correlations with multiple metabolite groups such as lipids, terpenoids, carbohydrates and their derivatives, and phenolic acids. However, these modules were mostly significantly negatively correlated with photosynthesis and growth-related indices including ribosome, steroids and their derivatives, chlorophyll, and antioxidant enzymes. This indicated that under defensive conditions, carbon flux shifted from growth-related pathways such as protein synthesis and steroid metabolism to defensive secondary metabolic pathways including cuticle and wax formation, and phenylpropanoid biosynthesis.

Furthermore, “Plant–pathogen interaction” showed a systematic negative correlation with indices such as ribosome, chlorophyll, CAT, and POD, but was closely positively correlated with phenylpropanoid and unclassified secondary metabolic pathways. This suggested that upon activation of pathogen-related signals, plants suppress growth processes such as photosynthesis and translation, while significantly inducing defense-related secondary metabolism and epidermal barrier construction.

## 4. Discussion

As an emerging contaminant, nanoplastics (NPs) have posed a significant threat to the growth and metabolism of aquatic plants, while the intervention of selenium nanoparticles (NPs-Se) provides an effective approach to alleviate this stress. Through an integrated analysis of physiological and biochemical assays, scanning electron icroscopy (SEM), confocal laser scanning microscopy (CLSM), and multi-omics techniques, combined with correlation network analysis of gene functions, metabolite groups, and physiological indices (Figure 8), this study systematically elucidated the response mechanism of *P. stratiotes* under NP stress and the regulatory pathway of NPs-Se.

Energy metabolism, as the core foundation of plant growth, development, and stress resistance, is susceptible to external stress disturbances. Transcriptomic data revealed that NP stress significantly inhibited the function of the ribosome pathway (map03010), which is consistent with the findings of studies on rice, cabbage, and other plants under microplastic stress [22,23]. As the core site of protein synthesis, impaired ribosome function directly reduces the synthesis of key enzymes in energy metabolism pathways such as glycolysis, thereby interfering with energy conversion efficiency. Further correlation network analysis confirmed that the ribosome module exhibited a significant positive correlation with chlorophyll, CAT, and POD indices, indicating that decreased ribosome activity would synchronously impair the functions of the photosynthetic and antioxidant systems. Meanwhile, it showed a significant positive correlation with metabolite groups involved in cutin, suberin, and wax biosynthesis, phenylpropanoid biosynthesis, lipids, and terpenoids, but a negative correlation with growth-related indices such as ribosome and chlorophyll. This suggests that under NP stress, carbon flux shifts from growth-related pathways to defensive secondary metabolic pathways (Figure 8). NP stress activates RING-type E3 ligases in the ubiquitin-proteasome system (UPS), mediating the ubiquitin-dependent degradation of ribosome-related regulatory proteins and further exacerbating ribosome dysfunction [24], which aligns with the classic mechanism by which UPS regulates metabolic pathways through protein degradation in plant stress responses. After NPs-Se treatment, the function of the ribosome pathway was significantly restored, presumably by repairing the protein synthesis system to improve energy metabolism efficiency. NPs-Se can regulate the expression of genes involved in starch and sucrose metabolism to optimize carbohydrate allocation [25]. In this study, the continuous upregulation of the motor protein pathway further mediates organelle transport and energy substance translocation, forming a positive feedback loop with the ribosome pathway [26]. More importantly, NPs-Se maintains ribosome functional stability by regulating the activity of the 26S proteasome α2, β5 subunits in the UPS, inhibiting the abnormal ubiquitin-mediated degradation of ribosomal proteins while promoting the selective clearance of damaged proteins [22]. Metabolomic data showed that the content of adenine, a key metabolite in energy metabolism, was significantly decreased under NP stress, while NPs-Se treatment effectively restored its abundance (Figure 7). Combined with the positive correlation between ribosomes and energy metabolites in the correlation analysis, these results indicate that NPs-Se synergistically alleviates the energy metabolism disorders caused by NPs at both the transcriptional and metabolic levels, providing energy support for the stress resistance of *P. stratiotes* (Figure 8).

The antioxidant system, as a key barrier against reactive oxygen species (ROS) and oxidative damage, is significantly activated under NP stress. This study found that NP stress activates the phenylpropanoid biosynthesis pathway, promoting the synthesis of phenolic acids and flavonoids through phenylalanine ammonia-lyase (PAL) and 4-coumarate-CoA ligase (4CL), which synergize with GSH to scavenge ROS [26,27]. SEM observations further confirmed that NP stress leads to cell membrane rupture and cell wall damage, while CLSM showed massive accumulation of NPs in root and stem tissues, as well as vascular and cortical tissues, exacerbating oxidative stress damage. Under NP stress, the UPS mediates the turnover of key proteins in the antioxidant system through U-box E3 ligases, promoting the ubiquitin-dependent degradation of excessively oxidatively damaged proteins while regulating the activities of CAT and POD [28]. NPs-Se treatment significantly enhanced the antioxidant capacity of *P. stratiotes*, characterized by increased CAT and POD activities and GSH content. This effect is attributed to the activation of glutathione peroxidase (GSH-PX) synthesis by NPs-Se and the regulation of the ferroptosis resistance pathway mediated by the fgf21 gene [29]. Notably, NPs-Se does not simply amplify the defense response but achieves metabolic recalibration by reshaping the flux of the phenylpropanoid pathway [15]. SEM and CLSM observations showed that after NPs-Se treatment, cell membrane integrity was restored, cell arrangement was compact, NP accumulation was reduced, and ultrastructural damage to root and stem tissues was alleviated, confirming the effective mitigation of oxidative damage. This is consistent with studies on lettuce and pepper, where NPs-Se promotes lignin and phenolic accumulation by regulating the phenylpropanoid pathway [30,31].

Plant hormones mediate stress responses through complex regulatory networks. Under NP stress, the plant-pathogen interaction pathway was significantly enriched. As a marker of immune defense activation [32], the coordinated enrichment of this pathway with the phenylpropanoid pathway implies cross-regulation between hormone signals and secondary metabolic defense. Further correlation network analysis revealed that the plant-pathogen interaction module exhibited a systematic negative correlation with growth-related indices such as ribosome and chlorophyll, but a close positive correlation with phenylpropanoid metabolism. This indicates that NP-induced signals inhibit photosynthesis and translation processes, prioritizing the activation of defensive secondary metabolism and epidermal barrier construction (Figure 8). CLSM observations showed that NPs accumulate in the vascular tissues of *P. stratiotes* roots and stems and migrate upward, potentially blocking substance transport and further exacerbating hormone signal disorders. NP stress can mediate the ubiquitin-dependent degradation of JAZ repressors in the jasmonic acid (JA) signaling pathway through the SCF-type E3 ligase complex, activating the JA defense response; however, excessive activation leads to growth inhibition [33] NPs-Se treatment optimizes stress responses by regulating the hormone signal network. Studies on soybean seedlings have shown that high-dose NPs-Se can enrich the plant-pathogen interaction pathway and regulate auxin and salicylic acid (SA) signal transduction [34], which is consistent with the NP stress mitigation effect of NPs-Se in this study. In addition, the MYB96 transcription factor activates wax synthesis genes and SA biosynthesis, achieving coordination between hormone signals and structural defense [35]. NPs-Se moderately degrades excessively activated JA signaling components by regulating RING-type E3 ligase activity, avoiding growth inhibition caused by over-defense, while stabilizing auxin signal-related proteins to balance growth and defense [36]. Metabolomic data showed that JA content was significantly increased under NP stress, while NPs-Se moderately adjusted its abundance to prevent over-activation of defense from hindering growth, reflecting a precise regulatory effect. SEM observations showed that the cell wall structure of *P. stratiotes* was intact after NPs-Se treatment, further confirming the restoration of epidermal barrier function under hormone signal regulation.

Photosynthesis, as the core of plant material synthesis and energy conversion, is a sensitive target of NP stress. This study found that NP stress significantly reduced chlorophyll content in *P. stratiotes* and inhibited the function of the photosynthesis antenna protein pathway, which is consistent with the classic inhibitory mechanism of microplastic stress [33]. Genes of the LHCA/LHCB family in this pathway, as the core of the light-harvesting complex, have their expression inhibited, directly impairing photon absorption and energy transfer efficiency [23]. NP stress exacerbates photosynthetic apparatus damage by activating E3 ligases to mediate the ubiquitin-dependent degradation of key photosynthetic system proteins [37]. NPs-Se treatment effectively alleviated photosynthetic inhibition, significantly restoring chlorophyll content and the expression of photosynthesis-related genes. This effect is consistent with the photosynthetic protective role of NPs-Se in rice and cabbage, namely, enhancing the photochemical efficiency of PSII by upregulating the expression of core photosystem genes such as psbQ and psaG [19].

## 5. Conclusions

NP stress exerts its toxic effects by inhibiting ribosome function and the photosynthetic system, while activating oxidative stress and defensive secondary metabolism. In contrast, NPs-Se achieves stress mitigation through multi-dimensional regulation: at the energy metabolism level, it repairs the synergistic network of protein synthesis and substance transport; at the antioxidant level, it enhances the synergistic effect of enzymatic and non-enzymatic defense systems; at the hormone signaling level, it optimizes the cross-regulation between growth and defense; and at the photosynthesis level, it protects the photosynthetic apparatus and light energy utilization efficiency.

Observations from SEM and CLSM provide intuitive ultrastructural evidence for these mechanisms, while correlation network analysis reveals the modular synergy and trade-off relationships among genes, metabolites, and physiological indices. Together, these findings construct a comprehensive regulatory network mediated by NPs-Se for *P. stratiotes* against NP stress, offering theoretical support and practical references for the ecological remediation of water bodies contaminated by nanoplastics.

## 6. Environmental Implication

This study highlights the substantial environmental risks of nanoplastics (NPs) in aquatic ecosystems, as evidenced by their toxic effects on the growth and metabolism of *P. stratiotes*, a widespread floating aquatic plant. The distinct physiological, metabolic, and molecular responses of *P. stratiotes* to NP stress underscore its potential as a reliable bioindicator for NP pollution monitoring. Importantly, the multi-dimensional mitigation efficacy of selenium nanoparticles (NPs-Se) via regulating plant intrinsic stress resistance pathways offers a sustainable, eco-friendly strategy for remediating NP-contaminated water bodies, avoiding secondary pollution associated with traditional physical or chemical methods. These findings advance our understanding of NP-induced ecological perturbations and emphasize the need to incorporate NP stress and its mitigation mechanisms into aquatic ecological risk assessment, pollution management, and freshwater ecosystem protection strategies.

## Data Availability

The original contributions presented in this study are included in the article and Supplementary Materials. Further inquiries can be directed to the corresponding author.

## Supplementary Materials

The following supporting information can be downloaded at https://www.mdpi.com/article/10.3390/toxics14030244/s1, Table S1: Differentially Expressed Genes; Table S2: KEGG significant pathways; Table S3: Differential Metabolites.
