# Supplementary material for: Revealing the Selenium-Mediated Regulatory Mechanisms of P. stratiotes in Response to Nanoplastics Stress from Multiple Perspectives of Transcriptomics, Metabolomics, and Plant Physiology
Source: Toxics. 2026 Mar 11;14(3):244. doi: 10.3390/toxics14030244 (PMC13029896; doi:10.3390/toxics14030244)
Supplement: Supplementary file 1 [file toxics-14-00244-s001.zip › toxics-4145649-supplementary.pdf]

Supplementary Material

Table S1 Differentially Expressed Genes
Table S2 KEGG significant pathways
Table S3 Differential Metabolites

Table S1

Gene_id	Index	NR description	CK_1	CK_2	CK_3	Ps_1	Ps_2	Ps_3
TRINITY_DN100116_c1_g1	10139	------	0.95	1.43	0.99	0.41	0.34	0.33
TRINITY_DN10013_c0_g3	10157	hypothetical protein LUZ60_004911 [Juncus effusus])	0	0	0.2	0.75	1.26	0.6
TRINITY_DN100163_c0_g4	10171	------	0	0	0	0.07	0.04	0.07
TRINITY_DN10038_c1_g1	10323	unnamed protein product [Spirodela intermedia])	0.07	0	0	0.98	0.79	0.91
TRINITY_DN100414_c0_g1	10344	predicted protein [Hordeum vulgare subsp. vulgare])	4.08	6.31	4.58	1.59	2.84	1.9
TRINITY_DN10044_c0_g1	10358	translationally controlled tumor protein [Hibiscus trionum])	0.5	0	0.26	4.78	5.43	4.35
TRINITY_DN10073_c0_g1	10490	------	2.78	3.43	3.26	0.93	0.75	1.12
TRINITY_DN100751_c0_g1	10498	hypothetical protein M9H77_05560 [Catharanthus roseus])	0	0.04	0.08	0.82	0.64	0.98
TRINITY_DN10115_c0_g1	10697	hypothetical protein TIFTF001_018498 [Ficus carica])	6.12	5.25	6.71	14.48	13.68	13.61
TRINITY_DN101197_c0_g1	10723	------	0	0	0	0.51	0.67	0.75
TRINITY_DN10130_c0_g2	10798	40S ribosomal protein S9-2 [Rosa chinensis])	1.2	1.44	1.79	0.5	0.37	0.53

Gene_id	Index	NR description	CK_1	CK_2	CK_3	Ps_1	Ps_2	Ps_3
TRINITY_DN101833_c0_g1	11075	endo-1,3;,4-beta-D-glucanase [Vigna angularis])	0.46	0.35	0.35	1.1	0.92	0.83

Table S2

ID	Description	GeneRatio	GeneRatio2
map03010	Ribosome	177/687	0.257641921
map04814	Motor proteins	37/687	0.053857351
map04626	Plant–pathogen interaction	26/687	0.037845706
map00196	Photosynthesis – antenna proteins	22/687	0.03202329
map00940	Phenylpropanoid biosynthesis	10/687	0.014556041
map00999	Biosynthesis of secondary metabolites — unclassified	7/687	0.010189229
map00073	Cutin, suberine and wax biosynthesis	4/687	0.005822416
map03010	Ribosome	177/687	0.257641921
map04814	Motor proteins	37/687	0.053857351
map04626	Plant–pathogen interaction	26/687	0.037845706
map00196	Photosynthesis – antenna proteins	22/687	0.03202329

Table S3

Metabolite	CK_1	CK_2	CK_3	Ps_1	Ps_2	Ps_3
Others	21.51636242	21.60893869	21.5478341	22.36545192	22.40302495	22.36492939
Lipids	12.97343523	12.92401163	13.02121655	13.26296521	13.23396949	13.30150356
Nucleotides and derivatives	7.154799966	7.051132432	7.098419546	7.308522638	7.359638648	7.275908811
Terpenoids	7.397424265	7.422741532	7.412188805	7.888040497	7.865379177	7.907937256
Carbohydrates and derivatives	4.93764217	4.940827639	4.938458455	4.964285905	4.968578727	4.977664504
Phenolic acids and derivatives	2.667611168	2.778331304	2.764674272	4.013360552	4.034438759	4.039577835
Steroids and steroid derivatives	4.654674703	4.66883147	4.711459583	4.172965869	4.162560804	4.155011004
Others	21.51636242	21.60893869	21.5478341	22.36545192	22.40302495	22.36492939
Lipids	12.97343523	12.92401163	13.02121655	13.26296521	13.23396949	13.30150356
Nucleotides and derivatives	7.154799966	7.051132432	7.098419546	7.308522638	7.359638648	7.275908811
